# From teeth to brain: dental caries causally affects the cortical thickness of the banks of the superior temporal sulcus

**DOI:** 10.1186/s12903-024-03899-2

**Published:** 2024-01-23

**Authors:** Mengqiao Wang, Ziyao Wang, Yajie Yu, Delu Zhao, Zhiyuan Shen, Fulan Wei

**Affiliations:** 1 Department of Orthodontics, School and Hospital of Stomatology, Cheeloo College of Medicine, Shandong University, Jinan, China; 2 Department of Oral and Maxillofacial Surgery, School and Hospital of Stomatology, Cheeloo College of Medicine, Shandong University, Jinan, China; 3 Shandong Key Laboratory of Oral Tissue Regeneration & Shandong Engineering Research Center of Dental Materials and Oral Tissue Regeneration & Shandong Provincial Clinical Research Center for Oral Diseases, Jinan, China; 4 National Clinical Research Center for Mental Disorders, Peking University Sixth Hospital/Institute of Mental Health, The Key Laboratory of Mental Health, Ministry of Health, Peking University, Beijing, 100191 China

**Keywords:** Dental caries, Brain cortex, Mendelian randomization analysis, Banks of the superior temporal sulcus, Tooth-brain axis, Alzheimer’s disease

## Abstract

**Objectives:**

Dental caries is one of the most prevalent oral diseases and causes of tooth loss. Cross-sectional studies observed epidemiological associations between dental caries and brain degeneration disorders, while it is unknown whether dental caries causally affect the cerebral structures. This study tested whether genetically proxied DMFS (the sum of Decayed, Missing, and Filled tooth Surfaces) causally impacts the brain cortical structure using Mendelian randomization (MR).

**Methods:**

The summary-level GWAS meta-analysis data from the GLIDE consortium were used for DMFS, including 26,792 participants. ENIGMA (Enhancing NeuroImaging Genetics through Meta Analysis) consortium GWAS summary data of 51,665 patients were used for brain structure. This study estimated the causal effects of DMFS on the surface area (SA) and thickness (TH) of the global cortex and functional cortical regions accessed by magnetic resonance imaging (MRI). Inverse-variance weighted (IVW) was used as the primary estimate, the MR pleiotropy residual sum and outlier (MR-PRESSO), the MR-Egger intercept test, and leave-one-out analyses were used to examine the potential horizontal pleiotropy.

**Results:**

Genetically proxied DMFS decreases the TH of the banks of the superior temporal sulcus (BANSSTS) with or without global weighted (weighted, β = − 0.0277 mm, 95% CI: − 0.0470 mm to − 0.0085 mm, *P* = 0.0047; unweighted, β = − 0.0311 mm, 95% CI: − 0.0609 mm to − 0.0012 mm, *P* = 0.0412). The causal associations were robust in various sensitivity analyses.

**Conclusions:**

Dental caries causally decrease the cerebral cortical thickness of the BANKSSTS, a cerebral cortical region crucial for language-related functions, and is the most affected brain region in Alzheimer’s disease. This investigation provides the first evidence that dental caries causally affects brain structure, proving the existence of teeth-brain axes. This study also suggested that clinicians should highlight the causal effects of dental caries on brain disorders during the diagnosis and treatments, the cortical thickness of BANKSSTS is a promising diagnostic measurement for dental caries-related brain degeneration.

**Supplementary Information:**

The online version contains supplementary material available at 10.1186/s12903-024-03899-2.

## Introduction

Dental caries is one of the most common oral diseases that affects 28.7% of the global population [1]. Besides its local impacts such as pulpitis, tooth loss, and declined mastication, it might also affect distal organs [2]. In mice with declined mastication, a significant decrease was observed in synaptic density and the number of neurons [3], indicating that masticatory movements are important for preventing neurodegeneration. Tooth loss is relative to cognitive impairment and the risk of dementia [4–6]. Though dental caries is one of the most common causes of tooth loss and occlusion collapse [2], there have been no studies revealing the causal relationship between dental caries and brain alterations.

Recognizing the possible causal association of dental caries on the brain structure changes and identifying specific cerebral regions affected by dental caries could provide an effective approach for the early diagnosis of dental caries-related brain disorders. However, to date, no available evidence has revealed the association between dental caries and brain cortical structures.

Magnetic Resonance Imaging (MRI) is a sensitive tool to identify alterations in brain cortical structures [7, 8]. Mendelian randomization (MR) is a method using genetic proxies of exposure to infer the causal relationships between risk factors and diseases, overcoming confounding biases present in observational studies [9]. Two-sample MR analysis was an extension of the MR method, which realizes the utilization of summary statistics of genome-wide association studies (GWASs) in MR studies. For this investigation, a two-sample MR analysis was conducted to examine whether there are causal impacts of dental caries on the brain cortical structures. Specifically, this study employed the DMFS (the sum of Decayed, Missing and Filled tooth Surfaces) index as the measurement for dental caries and focused on the cortical thickness (TH) and surface area (SA) of the human cerebral cortex, which were identified through the use of MRI.

The objective of this study was to use MR to demonstrate the causal impacts of dental caries on the cerebral cortical SA and TH assessed from MRI, thereby providing new perspectives on the potential existence of a teeth-brain axis and uncovering the causal association between dental caries and brain structures/disorders.

## Materials and methods

This study utilized publicly accessible GWAS summary data. The research was conducted following the STROBE-MR statement [10].

### Data sources for instrumental variables of DMFS

The summary-level GWAS data correlated with DMFS was obtained from a meta-analysis of the GWAS from the GLIDE consortium (*n* = 26,792) [11]. This meta-analysis of GWAS contains 14,976 participants of European ancestry from 8 studies and 11,816 participants of Hispanic/Latino background. Genetic effect estimates in the DMFS meta-analysis were compared between studies of HCHS/SOL and other European ancestry for lead-associated single variants. There was little evidence for heterogeneity (PFDR ≥0.05 in all tests for heterogeneity). All participants provided samples for genetic analysis and were clinically assessed for the DMFS. DMFS in each included cohort is derived from clinical dental records. The details of the participants of DMFS from the GLIDE Consortium are reported in Table S1.

### Data origin for SA and TH of cerebral cortex

The GWAS data for brain cortical structure were acquired from the ENIGMA (Enhancing NeuroImaging Genetics through Meta Analysis) Consortium [8], which is a meta-analysis of GWAS conducted on 51,665 individuals. These individuals had their brain cortical TH and SA data measured using whole-brain T1-weighted MRI scans. This study utilized the GWAS meta-analysis findings from 33,992 individuals of European descent. Among these, 23,909 individuals were part of the ENIGMA consortium, representing 49 cohorts, while 10,083 individuals were sourced from the UK Biobank. Detailed information about the cohorts can be found in Table S2. The structure of the cerebral cortex was evident through the measurement of cerebral cortex TH and SA, which were calculated for each person throughout the entire cortex and within 34 specific regions defined by gyri, following the Desikan-Killiany atlas [12] and were calculated as the average of the regions in both hemispheres. MR analysis was conducted from DMFS to the entire cortex, including TH and SA, as well as 34 cerebral cortical regions with established functional specializations. The analysis for functional regions was performed on SA and TH with estimates of the global brain or not, resulting in a total of 136 outcomes. The weighted SA and TH on a global scale represent particular areas throughout the entire brain, whereas those without global weighting demonstrate the SA and TH measurement of specific regions irrespective of the overall SA and TH of the brain.

### Selection of genetic instruments

Genetic instruments of DMFS were selected according to the following criteria: a GWAS-correlated *P*-value of 5 × 10^−8^, a linkage disequilibrium [LD] r^2^ < 0.001, and within 1 MB distance from the index variant.

### Mendelian randomization analyses

The primary method used for the MR analysis to examine the impact of DMFS on brain structure was the random effects inverse-variance weighted (IVW) model. The significant *P*-value was set as 0.05. The other four models, MR Egger, Weighted median, Simple mode, and Weighted mode were employed for supplementary analysis. For significant estimates derived from the IVW model, if the estimates from other approaches were inconsistent, a tightened instrument P-value threshold would be applied and then the MR analysis was re-performed. For significant estimates, to additionally investigate potential horizontal pleiotropy, the MR pleiotropy residual sum and outlier (MR-PRESSO), MR-Egger intercept test, and leave-one-out analyses were performed. MR analysis was re-performed after removing the outlier SNPs identified using MR-PRESSO. Additionally, Cochran’s Q test was employed to detect the heterogeneity of IVs. To evaluate the probable directional pleiotropy, a funnel plot was employed. PhenoScanner (www.phenoscanner.medschl.cam.ac.uk), an extensive platform providing detailed data on the correlation between genotype and phenotype, was utilized additionally to detect and exclude the SNPs linked to potential risk factors associated with cortical composition. This was done to account for the possibility that IVs impact cortical structures via alternative pathways rather than DMFS. All MR analyses were conducted using the “TwoSampleMR (0.5.7)” packages, data visualizations were performed using “ggplot2”, “ggseg”, and “forestploter” packages within R software, specifically version 4.3.1.

## Results

**Fig. 1:**
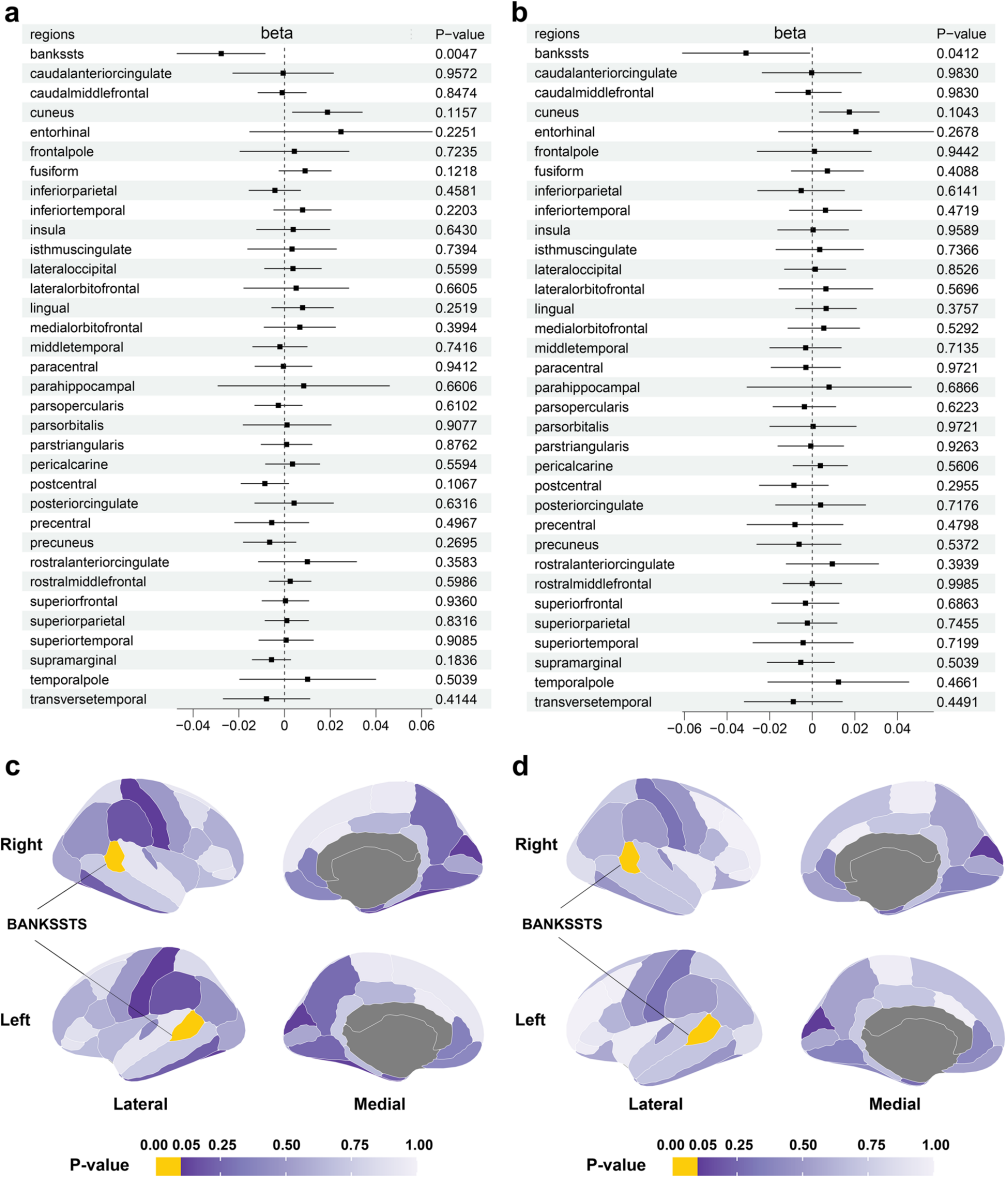
IVW-derived causal effects of DMFS on regional TH with (**a, c**) or without global weighted (**b, d**). (**a, b**) Forest plots show 95%CI of causal-effect estimates DMFS on the TH of 34 functional cerebral regions, the TH of the BANKSSTS was significantly impacted (weighted, β = − 0.0277 mm, 95% CI: − 0.0470 mm to − 0.0085 mm, P = 0.0047; unweighted, β = − 0.0311 mm, 95% CI: − 0.0609 mm to − 0.0012 mm, P = 0.0412). (**b, d**) Heatmaps show *P*-values of causal-effect estimates DMFS on the TH of 34 functional cerebral regions

**Fig. 2:**
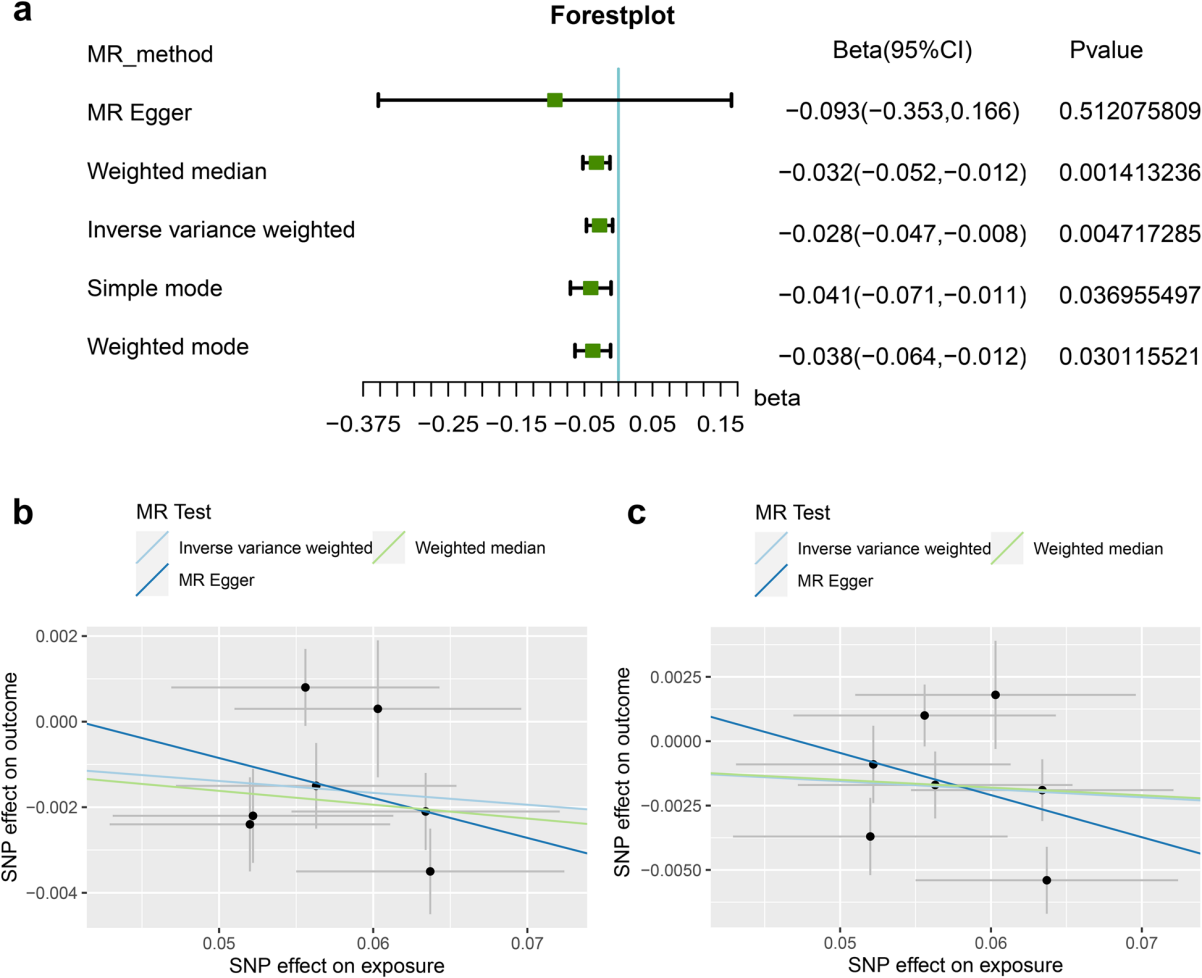
Causal effects of DMFS on the TH of the BANKSSTS. **a** ( Forest plot elucidates the declined TH of BANKSSTS induced by DMFS, estimated by 5 methods, Weighted median, IVW, Simple mode, and Weighted mode illustrated significant decreasing effects (*P* < 0.05), MR-Egger concurrently demonstrated the decreasing effect on the TH of BANKSSTS. Scatter plots of the associations of IVs with the TH of BANKSSTS with (**b**) or without global weighted (**c**)

Nine index SNPs, rs165383, rs622430, rs668415, rs2621287, rs3112580, rs3755402, rs4744611, rs11699974, and rs12092971, associated with DMFS (*P* < 5 × 10^−8^) and passed the linkage disequilibrium [LD] r^2^ < 0.001, and < 1 MB distance from the index variant were found in the ENIGMA GWAS, and therefore used as IVs (Table 1). This study conducted MR-analysis from the genetically predicted DMFS on the SA and TH of both the entire and regional cortex (Table S3-S4). At the global level, DMFS did not have a causal impact on the SA or TH of the entire cerebral cortex (Table S3). As for the regional SA, none was detected to be significantly affected by genetically predicted DMFS (Fig. S1, Table S4, S7–8). As for the functional region-level analysis of TH, as shown in Fig. 1 and Table 2, S5–6, IVW analysis detected that DMFS significantly decreased the TH of the Banks of the Superior Temporal Sulcus (BANKSSTS) with global weighted (β = − 0.0277 mm, 95% CI: − 0.0470 mm to − 0.0085 mm, *P* = 0.0047) and BANKSSTS without global weighted (β = − 0.0311 mm, 95% CI: − 0.0609 mm to − 0.0012 mm, *P* = 0.0412). Fig. 2a and Fig. S2 show forest plots of the estimates for TH of globally weighted BANKSSTS and TH of BANKSSTS without global weighted using the different MR methods, respectively, the detailed data was shown in Table S10. As shown in Fig. 2a, results derived from Weighted median, Simple mode, and Weighted mode-derived further elucidated the declined TH of BANKSSTS induced by DMFS (*P* < 0.05), the estimate derived from MR-Egger was also in concurrent direction. As shown in Fig. S2, the beta estimates of DMFS on the TH of BANKSSTS without global estimates derived from five MR-analysis were all negative, corroborating the negative effects. Fig. 2b and c show scatter plots of the associations of IVs with the TH of BANKSSTS with or without global weighted, respectively.

**Table 1:** Detailed information of instrumental variables (IVs)

SNP	Effect Allele	Other Allele	eaf	Beta	SE	*P* value
rs165383	A	G	0.5643	0.0603	0.0093	1.07E-10
rs622430	C	G	0.4079	−0.0603	0.0089	1.11E-11
rs668415	T	C	0.4646	0.0522	0.0091	9.72E-09
rs2621287	A	G	0.5591	0.0634	0.0087	3.36E-13
rs3112580	A	G	0.5271	0.0556	0.0087	1.48E-10
rs3755402	T	C	0.5626	0.0637	0.0087	2.43E-13
rs4744611	T	C	0.5003	−0.0563	0.0091	4.97E-10
rs11699974	T	C	0.5404	−0.052	0.0092	1.58E-08
rs12092971	T	C	0.4652	0.052	0.0091	1.15E-08

**Table 2:** Mendelian randomization estimated effects from DMFS on genetically predicted cortical structure

Exposure	Outcome	IVW-derived *P* value	beta (SE)
DMFS	TH of BANKSSTS with global weighted	0.0047**	−0.0277 mm (0.0098 mm)
DMFS	TH of BANKSSTS without global weighted	0.0412*	−0.0311 mm (0.0152 mm)

**Significant that IVW-derived *P* < 0.01, * Significant that IVW-derived *P* < 0.05

*DMFS* Decayed-Missed-Filled-Surfaces: *BANKSSTS* Banks of the Superior Temporal Sulcus: *IVW* Inverse-Variance Weighted: *MR* Mendelian Randomization: *TH* cortical thickness: *SA* cortical surficial area: *SE* Standard Error

**Fig. 3:**
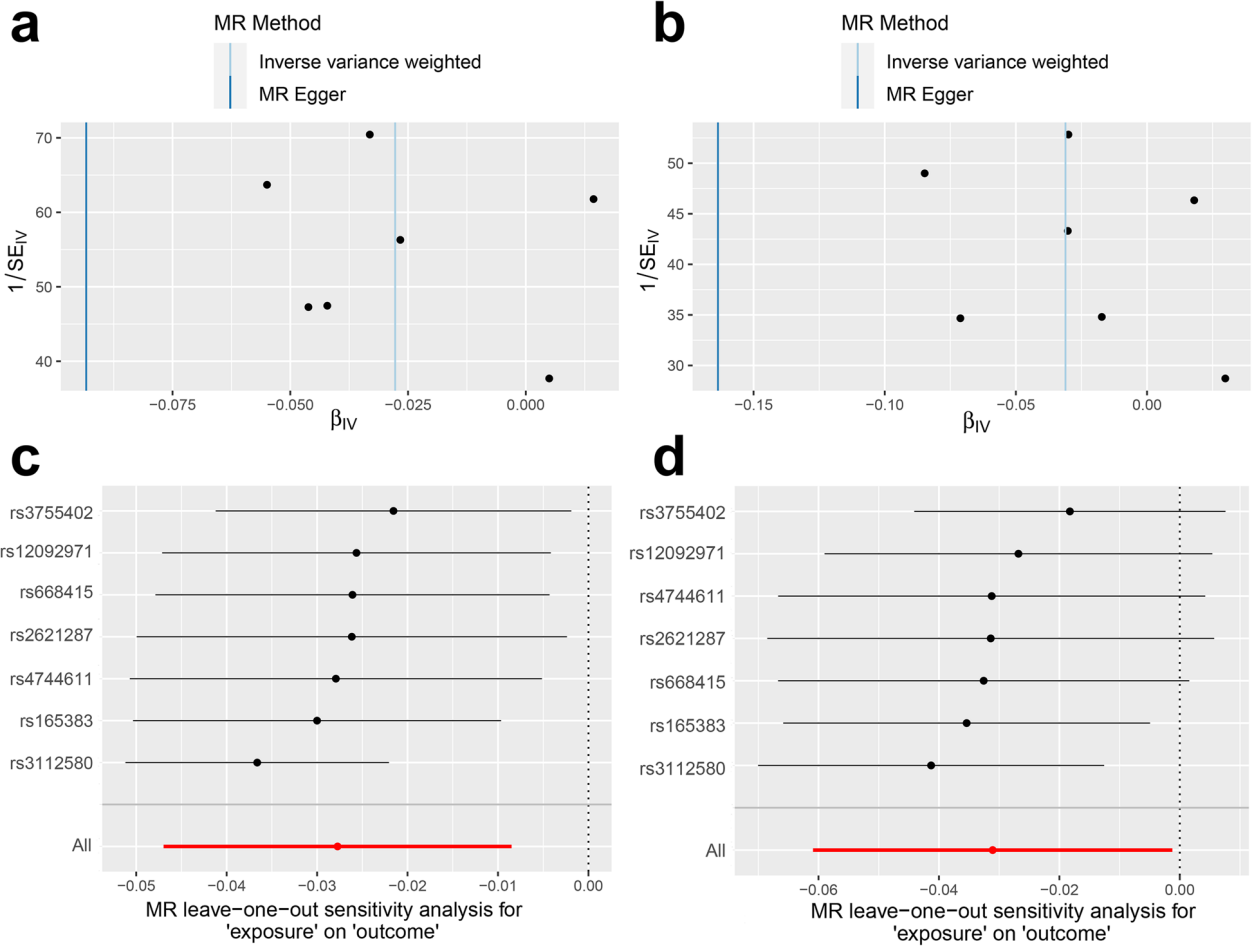
Funnel plots (**a, b**) and Leave-one-out analyses (**c, d**) of DMFS on the weighted (**a, c**) and unweighted (**b, d**) TH of the BANKSSTS

MR-Egger intercept test, Cochran’s Q test, funnel plots, and leave-one-out analyses were used to assess the horizontal pleiotropy of the significant estimates. MR-Egger intercept test indicates no significant possible horizontal pleiotropy, implying robust causal effects from genetically predicted DMFS on the TH of BANKSSTS. (*P* > 0.05, Table S10). Cochran’s Q test implies heterogeneity among the estimates of IVs (*P* < 0.05, Table S11). Leave-one-out analyses and funnel plots are provided in Fig. 3. As Fig. 3a and b show, the SNPs are distributed equally at two sides of IVW, indicating that the results of this study are relatively robust. As Fig. 3c shows, the 95% confidence interval (CI) is stable at the region below zero, indicating that the result that genetically predicted DMFS decrease the globally weighted TH of BANKSSTS cortex is robust when any IV was excluded. Consistently, most CI estimates the causal effect of DMFS on the TH of BANKSSTS without globally weighted located at the region below zero when any IV was excluded (Fig. 3d), consistently illustrating the negative effect of DMFS on the TH of BANKSSTS. Additionally, in the PhenoScanner search, there are no associations between IVs and previously reported traits, implying the effect of IVs on the TH on BANKSSTS was not through other previously found traits.

## Discussion

To the best of our knowledge, this is the first study that thoroughly establishes the causal impacts of dental caries on the structure of the cerebral cortex. The present MR study systematically assessed the causal effects of genetically predicted DMFS on the brain cortical structure, including SA and TH of the entire cortex and 34 functional cerebral cortical regions. This study demonstrated that dental caries causally decreased the thickness of the BANKSSTS, with no significant horizontal pleiotropy. This finding suggests the pathophysiologic connections from dental caries to the degeneration in the brain, thus emphasizing the presence of the teeth-brain axes.

Studies have suggested the axis from mouth to brain in recent years. Horinuki et al. detected sequential excitation in brain somatosensory and insular cortices responding to periodontal ligament (PDL) stimulation in the tooth movement model of rats [13]. In addition, the activities of neurons in the insular cortex changed during the tooth movement process [14], these results evidenced tooth-brain axes from activation of PDL to the neurons in the somatosensory and insular cortices. Moreover, the epidemiology association between cerebral palsy and dental caries in children also provides evidence for the presence of the tooth-brain axis [15]. Among the clinically estimated parameters for dental caries, DMFS has the highest heritability [15], making it the proper estimate for MR analysis of dental caries. Result of this study was in agreement with these findings demonstrated the presence of tooth-brain regulation axes, and provided a novel tooth-brain axis from dental caries to the banks of the superior temporal sulcus cortex.

The STS is a functional region definitely arranged by several components, rather than a single structure [16]. Previous meta-analyses of structural MRI studies, functional MRI studies, and positron emission tomography studies have detected a wide range of language- and reading-related functions in the STS, such as sublexical processing of speech [17–19] and representation of phonological word forms [20]. The decline in gray matter volume of the left STS was found associated with poor reading comprehension and reading disability [19], which further evidenced the importance of STS in reading-related functions. BANKSSTS is the region where the processing of written and spoken language converges, between modality-specific preprocessing and language comprehension [21–24]. A recent GWAS study with a sample size of 34,000 found that the BANKSSTS on the left cerebral hemisphere is the cerebral cortex region most associated with the processing of spoken and written language [25]. The current study demonstrated that genetically predicted DMFS causally decreased the thickness of the BANKSSTS, suggesting that increased teeth surface suffering from dental caries may related to declined reading- and language-related functions, while further functional studies are required to elucidate whether a functional alteration therein.

The presence of widespread cortical β-amyloid (Aβ) is the initial event that leads to AD, recent florbetapir-PET studies revealed that BANKSSTS is the highest Aβ-affected cortical region [26]. In favor, Aβ-burden in the BANKSSTS is a sensitive indicator for determining the prognosis of AD, individuals with higher Aβ-burden BANKSSTS exhibited faster rates of memory decline and executive function decline [26]. A longitudinal study also concurrently found that BANKSSTS, posterior cingulate cortex (PCC), and precuneus showed the fastest rates of Aβ accumulation at a very early stage in mutation carriers of autosomal dominant AD [27], and BANKSSTS was also among the earliest regions to show glucose hypometabolism and cortical atrophy. Numerous cross-sectional studies have found that patients with AD or dementia usually have poor oral conditions and a higher prevalence of tooth loss [28], which has been long considered a consequence of difficulty of oral care due to impaired cognitive function, memory, and physical ability in patients with dementia. However, recent studies have identified tooth loss to be a cause of brain degeneration and AD [4–6]. In edentulous subjects, a three-dimensional magnetic resonance imaging study found significant atrophy in the memory, learning, and cognition-related cortical regions, including the caudate nucleus, hippocampus, and temporal pole of the right hemisphere [29]. Tooth loss has also been regarded as an important risk factor for the onset of AD [30]. Though dental caries is one of the most common reasons for tooth loss [2], its impact on brain structure and function has not been elucidated. This study demonstrated that DMFS causally decreased the TH of BANKSSTS, therefore evidencing the causal impacts of dental caries on cerebral structure degeneration, and providing causal evidence supporting the causal effects of caries on AD.

This study has some limitations. First, the quantitative estimates using MR were based on a linear relationship between the exposure and the outcome; that may be some kind of misleading if the true relationship is nonlinear. Second, LD patterns can vary among different populations, thereby the causal connection between dental caries and the cerebral cortical structure in other ancestries remains uncertain. Third, this investigation only demonstrated the causal effects of dental caries on the cerebral cortical structure while its underlying mechanisms and functional alterations warrant further investigation.

Taken together, this is the first study revealing the causal effects of dental caries on cerebral cortical structures. Our findings detected that DMFS causally decreased the cortical thickness of the BANKSSTS, which is an important region for spoken and written language, and the most impaired cortical region in AD. This finding revealed a novel tooth-brain axis, that from dental caries to the BANKSSTS, and provided evidence supporting the dental caries-associated brain degeneration and disease. This study also suggested that clinicians should highlight the causal effects of dental caries on brain disorders during the diagnosis and treatments, the thickness of BANKSSTS might be a promising indicator for diagnosing dental caries-related brain degeneration.

### Supplementary Information

**Additional file 1.**

**Additional file 2.**

## Data Availability

No datasets were generated or analysed during the current study.
