# Supplementary material for: From teeth to brain: dental caries causally affects the cortical thickness of the banks of the superior temporal sulcus
Source: BMC Oral Health. 2024 Jan 23;24:124. doi: 10.1186/s12903-024-03899-2 (PMC10807149; doi:10.1186/s12903-024-03899-2)

**Supplementary Figures**

**Supplementary Figure 1.** IVW-derived causal effects of DMFS on regional SA with (a, c) or without global weighted (b, d). (a, b) Forest plots show 95%CI of causal-effect estimates DMFS on the SA of 34 functional cerebral regions, (b, d) Heatmaps show P-values of causal-effect estimates DMFS on the SA of 34 functional cerebral regions.

**Supplementary Figure 2.** MR-Egger, Weighted median, IVW, Simple mode, and Weighted mode estimated causal effects of DMFS on the TH of BANKSSTS without global estimate, corroborating the negative effects.


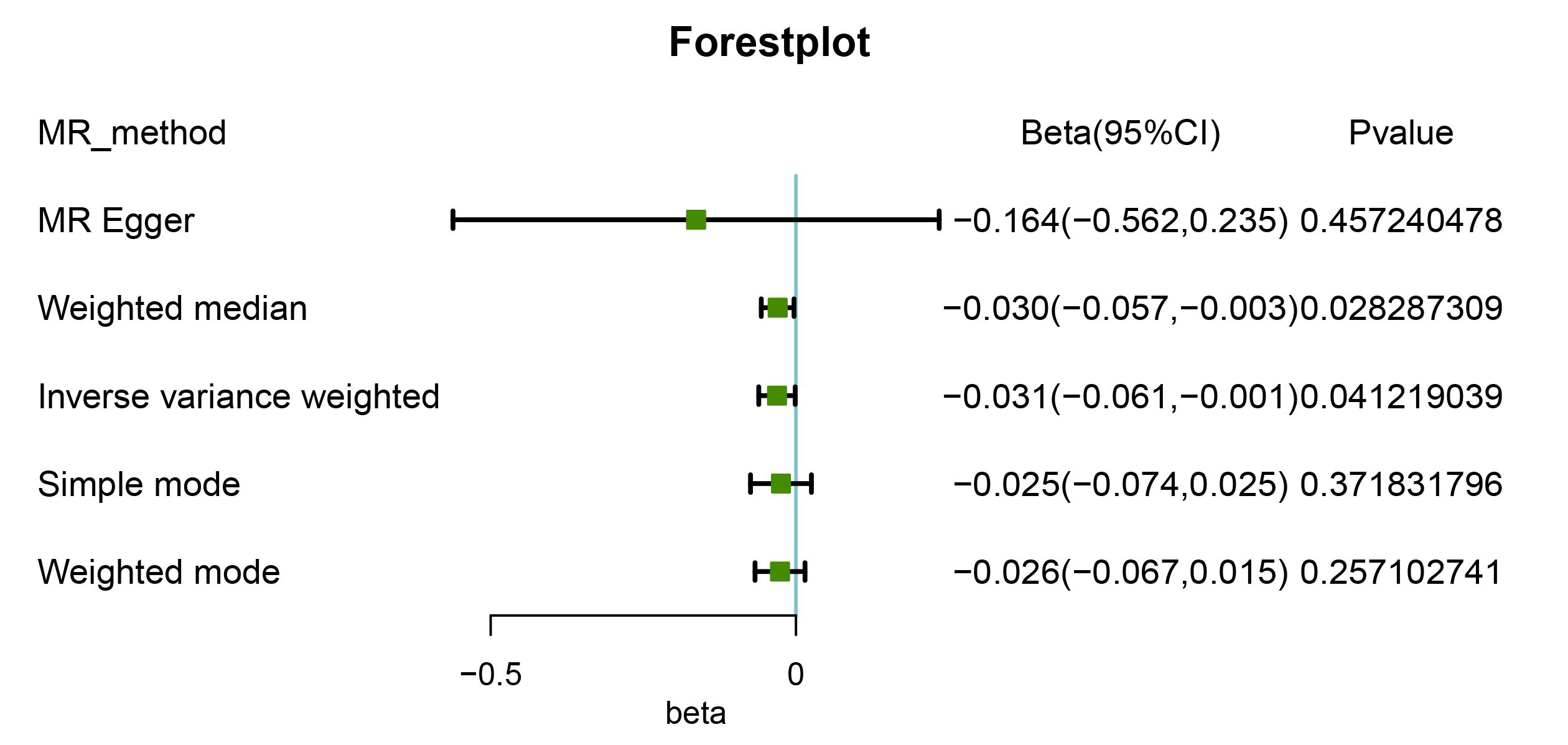

Supplement: Supplementary file 1 — Additional file 1. [file 12903_2024_3899_MOESM1_ESM.docx]
